# Andrographolide as a Promising Diterpenoid Scaffold Against the Neurotropic Nematode *Angiostrongylus cantonensis*

**DOI:** 10.3390/ph19091435

**Published:** 2026-09-10

**Authors:** Fabiana R. S. Tominaga, Davi Luna-Tavares, Lucas Fukui-Silva, Thainá R. Teixeira, Henrique Barbosa, João Henrique G. Lago, Josué de Moraes

**Affiliations:** 1 Research Center on Neglected Diseases, Brazil University, São Paulo 08230-030, SP, Brazil; fabitominaga@gmail.com (F.R.S.T.); davilunatav@gmail.com (D.L.-T.); 2 Research Center on Neglected Diseases, Guarulhos University, Guarulhos 07023-070, SP, Brazil; fukui.npdn@gmail.com (L.F.-S.); thainanpdn@gmail.com (T.R.T.); 3 Center for Natural and Human Sciences, Federal University of ABC, São Paulo 09210-180, SP, Brazil; heinrichbarbosa@gmail.com

**Keywords:** anthelmintic activity, *Angiostrongylus cantonensis*, andrographolide, *Cymbopogon schoenanthus*

## Abstract

**Background**: *Angiostrongylus cantonensis* is a neurotropic nematode recognized as the leading cause of eosinophilic meningitis worldwide. Despite its growing medical importance, therapeutic options for angiostrongyliasis remain limited, highlighting the need for new anthelmintic agents. **Methods**: The anthelmintic activity of andrographolide, a diterpene lactone isolated from *Cymbopogon schoenanthus*, was evaluated against first-stage (L1) larvae, infective third-stage (L3) larvae, and adult worms of *A. cantonensis* using motility-based phenotypic assays. Toxicity was assessed in mammalian cell models and *Caenorhabditis elegans*, and drug-likeness and ADMET properties were evaluated using in silico approaches. **Results**: Andrographolide exhibited concentration-dependent activity across all developmental stages, with EC_50_ values of 12.5, 11.2, and 7.3 µM for L1, L3, and adult worms, respectively. These EC_50_ values were similar to those of albendazole, with no statistically significant differences detected for any developmental stage. No toxicity was detected at the highest concentrations tested in mammalian cell models (CC_50_ > 500 µM) or *C. elegans* (LD_50_ > 1000 µM), supporting a preliminary selectivity profile. The in silico analyses predicted compliance with major drug-likeness rules, absence of PAINS alerts, high gastrointestinal absorption, and low probabilities of hepatotoxicity, nephrotoxicity, and neurotoxicity. **Conclusions**: Andrographolide represents a promising in vitro anthelmintic hit against *A. cantonensis* and warrants further preclinical investigation.

## 1. Introduction

Human angiostrongyliasis is an emerging zoonotic helminthiasis caused by nematodes of the genus *Angiostrongylus*. Among them, *Angiostrongylus cantonensis* is a neurotropic species recognized as the leading etiological agent of eosinophilic meningitis worldwide [1]. The parasite is widely distributed in tropical and subtropical regions, and its geographic expansion has been increasingly associated with globalization, climate change, urbanization, and the dissemination of invasive intermediate hosts [2,3]. Human infection occurs mainly through the ingestion of infective third-stage larvae (L3) present in contaminated mollusks, paratenic hosts, or vegetables, allowing larval migration to the central nervous system, where severe neurological manifestations may develop [4]. Beyond their clinical relevance as infective forms, L3 larvae have also become important experimental targets in anthelmintic drug discovery [5]. In parallel, first-stage larvae (L1) have increasingly been used as convenient phenotypic screening models for nematode drug discovery because of their accessibility, reproducibility, and suitability for high-throughput in vitro assays [6,7]. Adult worms also represent an important experimental stage in anthelmintic drug discovery, since most currently available anthelmintic drugs were developed against adult helminths [8,9]. Together, these developmental stages provide complementary experimental platforms for the identification and characterization of novel anti-nematode compounds.

Despite the widespread use of albendazole in the treatment of nematode infections [10], therapeutic efficacy against tissue-migrating larval stages remains inconsistent [11]. This scenario underscores the need for the discovery of novel anthelmintic compounds with improved efficacy. In this context, natural products continue to represent an important source of bioactive scaffolds for anthelmintic drug discovery [12,13]. Structurally diverse metabolites derived from medicinal plants have yielded promising compounds with broad pharmacological properties. Among them, diterpenoids have attracted considerable attention because of their anti-inflammatory, antioxidant, and antimicrobial activities [14], as well as their potential as lead molecules for neglected tropical disease drug discovery [15].

Andrographolide has emerged as a pharmacologically versatile natural product with relevant therapeutic potential [16]. Previous studies have demonstrated antiparasitic activity of andrographolide against protozoan parasites, including *Leishmania* spp. [17,18], *Trypanosoma cruzi* [19], *Trypanosoma brucei* [20], and *Plasmodium* spp. [21], as well as parasitic nematodes such as the bovine filarial worm *Setaria cervi* [22] and the human hookworm *Ancylostoma duodenale* [23]. In addition to its antiparasitic properties, andrographolide exhibits anti-inflammatory, antioxidant, mitochondrial-protective, and neuroprotective activities mediated by multiple molecular pathways [16]. Experimental evidence has also demonstrated that andrographolide can modulate blood–brain barrier integrity and exert protective effects on cerebral endothelial cells [24], an especially relevant feature considering the neurotropic nature of *A. cantonensis* infection.

Although andrographolide has demonstrated a broad range of pharmacological and antiparasitic activities, its activity against *A. cantonensis* has not yet been investigated. The need for new agents active against tissue-migrating nematode stages, together with the documented antiparasitic profile of andrographolide, provided the rationale for its evaluation against this parasite. As andrographolide had previously been identified by our group in *Cymbopogon schoenanthus* [19], this species was selected as the botanical source for obtaining the compound used in the present study. Accordingly, andrographolide was isolated from its leaves and evaluated for in vitro anthelmintic activity against first-stage (L1) larvae, infective third-stage (L3) larvae, and adult worms of *A. cantonensis*. In parallel, toxicity was assessed in mammalian cells and in the invertebrate model *Caenorhabditis elegans*, whereas in silico ADMET and drug-likeness analyses were performed to provide preliminary insights into the pharmacological profile of this natural diterpenoid.

## 2. Results

### 2.1. Isolation and Identification of Andrographolide

**Figure 1:**
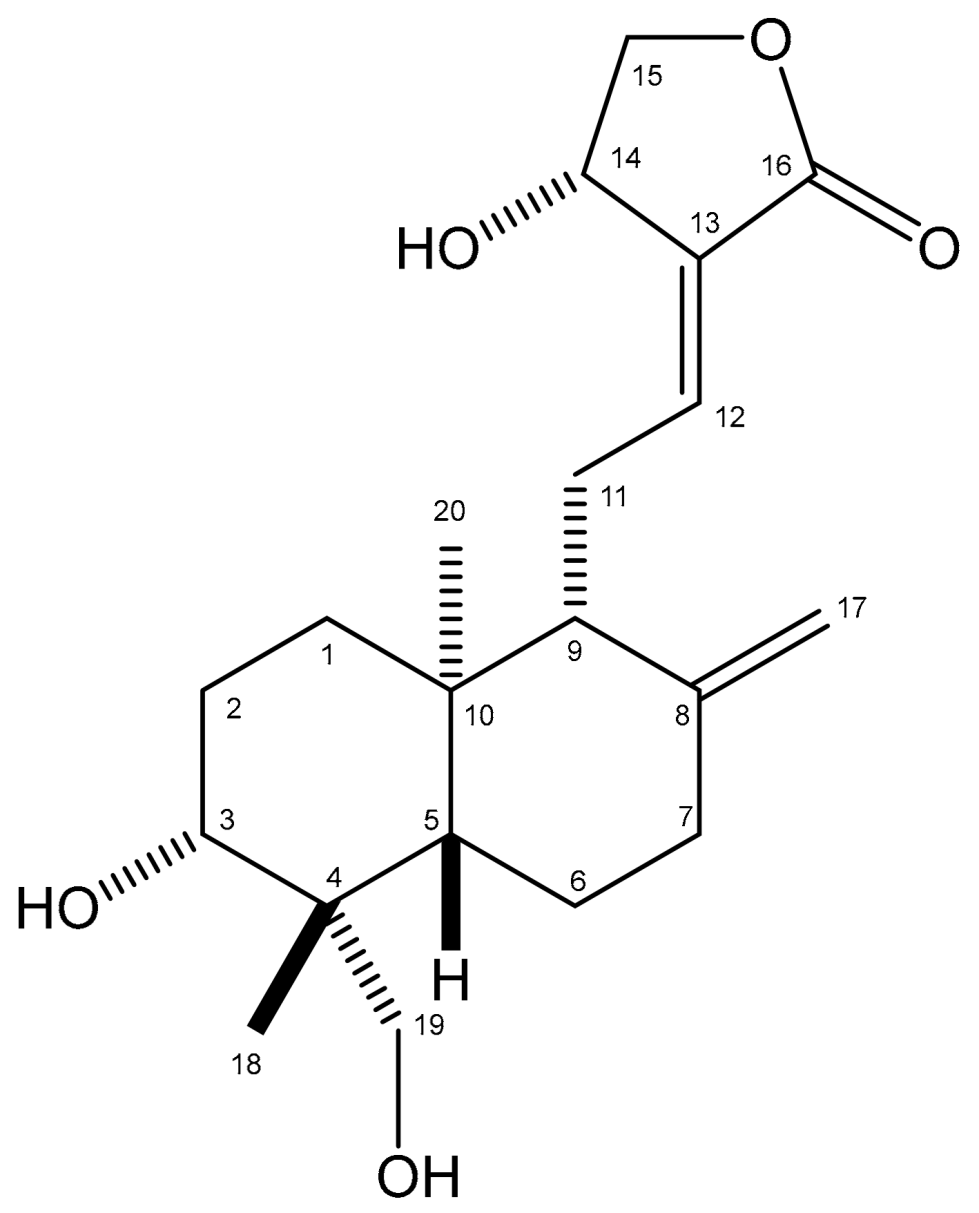
Chemical structure of andrographolide isolated from *C. schoenanthus*.

Dried leaves of *C. schoenanthus* were sequentially extracted with hexane, and the resulting extract was subjected to silica gel column chromatography. Further chromatographic purification afforded andrographolide as a white amorphous solid with 99% purity, as determined by HPLC analysis. Based on MS and NMR data, the isolated compound was identified as andrographolide (Figure 1), in agreement with previously reported data [25]. HPLC chromatogram and UV, ESI-HRMS, ^1^H and ^13^C NMR spectra are provided in the Supplementary Material.

### 2.2. Anthelmintic Activity Against Larval and Adult Stages of A. cantonensis

At the initial screening concentration of 100 µM, andrographolide exhibited marked anthelmintic activity against first-stage (L1) larvae, infective third-stage (L3) larvae, and adult worms of *A. cantonensis* after 24 h of incubation.

**Figure 2:**
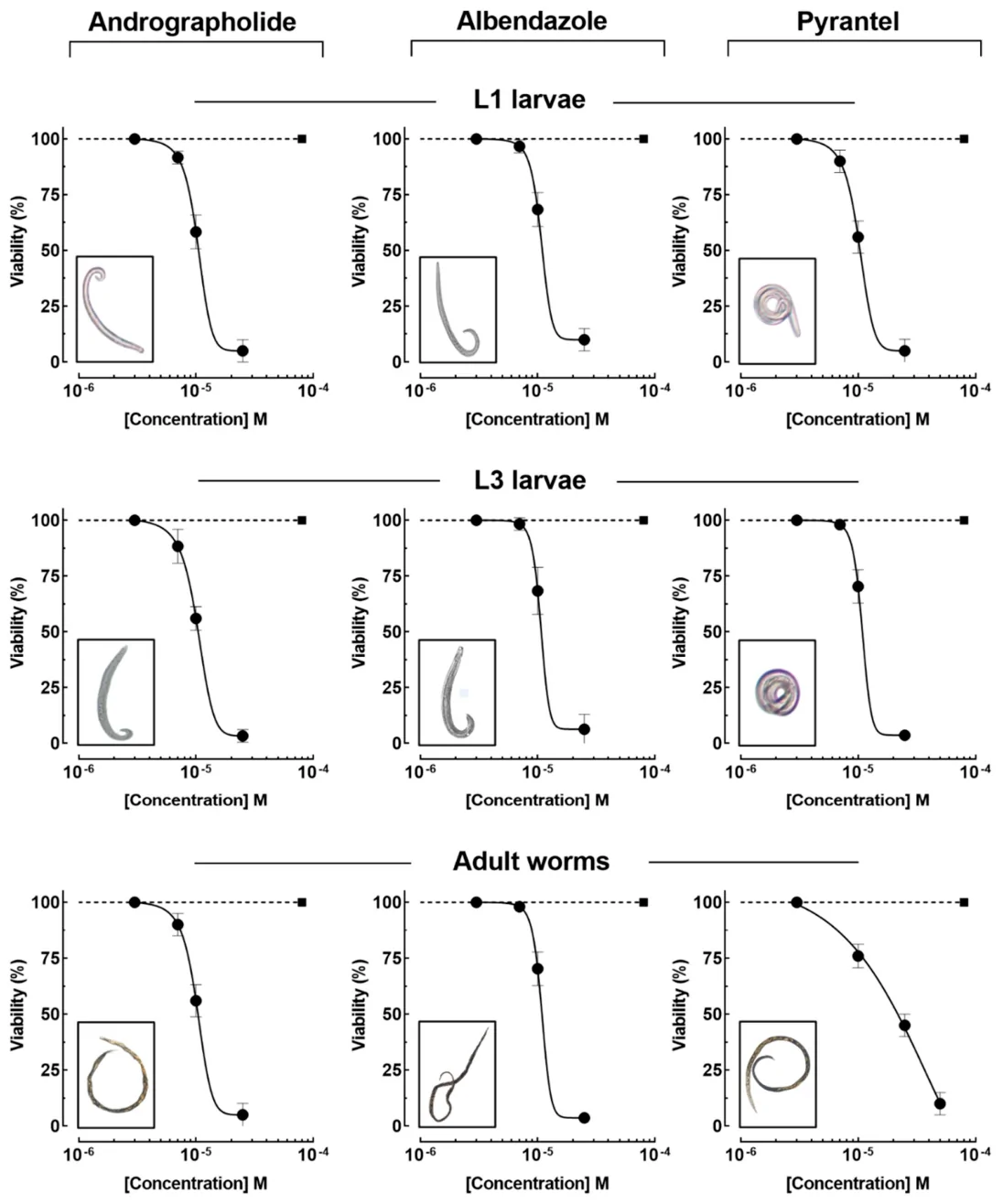
In vitro concentration–response curves of andrographolide, albendazole, and pyrantel against first-stage (L1) larvae, infective third-stage (L3) larvae, and adult worms of *A. cantonensis* after 24 h of incubation. Insets show representative elongated phenotypes following exposure to andrographolide or albendazole and the characteristic coiled phenotype induced by pyrantel. Data are presented as mean ± standard deviation (SD) from three independent experiments. Solid lines represent the nonlinear regression fits.

Subsequent concentration–response assays demonstrated a concentration-dependent reduction in parasite motility, with EC_50_ values of 12.5, 11.2, and 7.3 µM for L1, L3, and adult worms, respectively (Figure 2; Table 1). No statistically significant differences were detected among the three compounds against L1 or L3 larvae. Against adult worms, andrographolide did not differ significantly from albendazole, whereas pyrantel pamoate was significantly less potent than both compounds (*p* < 0.01).

Representative phenotypic alterations following drug exposure are shown in Figure 2. In both larval stages, pyrantel induced a markedly coiled phenotype, whereas andrographolide- and albendazole-treated parasites exhibited predominantly elongated phenotypes. Similar morphological alterations were observed in adult worms exposed to andrographolide and albendazole.

### 2.3. Toxicity Assessment in Mammalian Cells and C. elegans

The toxicity of andrographolide was evaluated in mammalian kidney epithelial (Vero) and neuronal (SH-SY5Y) cell lines, as well as in the invertebrate model *C. elegans*. No significant toxicity was observed in either mammalian cell line at the highest concentration tested, resulting in CC_50_ values > 500 µM. Likewise, 50% mortality was not reached in *C. elegans* at the highest concentration tested (1000 μM); therefore, the LD_50_ was reported as >1000 μM without extrapolation (Table 2). Based on these findings and the EC_50_ values obtained against *A. cantonensis*, andrographolide exhibited a preliminary selectivity profile, with minimum SI values of >40, >44, and >68 against L1, L3, and adult worms, respectively.

### 2.4. In Silico ADMET and Drug-Likeness Analysis

**Figure 3:**
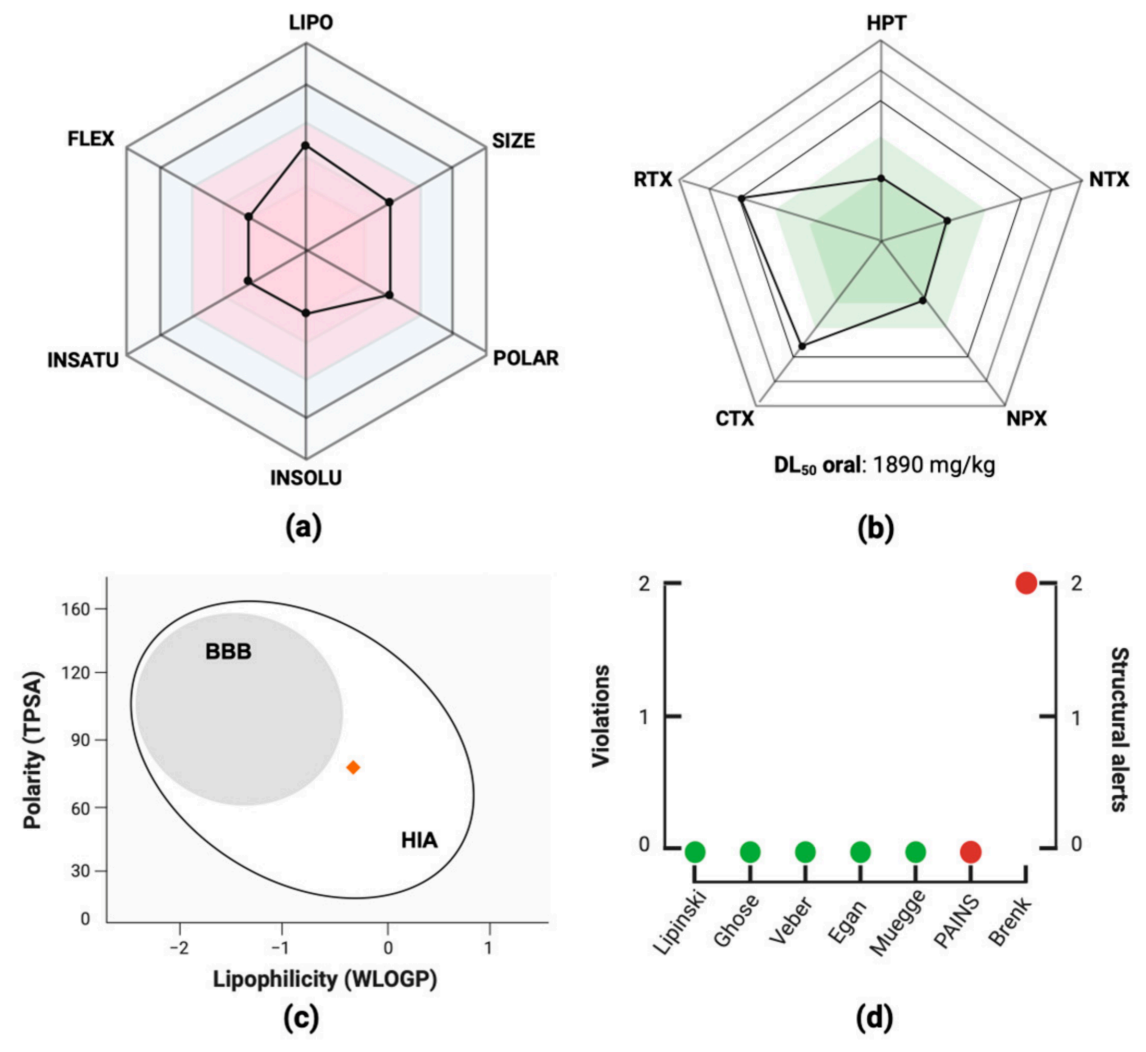
In silico ADMET and drug-likeness analysis of andrographolide. (**a**) Bioavailability radar showing physicochemical properties associated with oral drug-likeness, including lipophilicity (LIPO), molecular size (SIZE), polarity (POLAR), solubility (INSOLU), saturation (INSATU), and flexibility (FLEX). (**b**) Predicted organ toxicity profile, including hepatotoxicity (HPT), neurotoxicity (NTX), nephrotoxicity (NFX), cardiotoxicity (CTX), and respiratory toxicity (RTX). (**c**) BOILED-Egg model predicting gastrointestinal absorption (HIA) and blood–brain barrier permeability (BBB). (**d**) Drug-likeness rules and structural alert analysis, including Lipinski, Ghose, Veber, Egan, Muegge, PAINS, and Brenk filters.

In silico pharmacokinetic and drug-likeness analyses revealed favorable physicochemical characteristics for andrographolide (Figure 3). The compound showed no violations of the Lipinski, Ghose, Veber, Egan, or Muegge rules, supporting a drug-like profile compatible with oral bioavailability. In addition, andrographolide exhibited no PAINS alerts, whereas two Brenk alerts associated with the α,β-unsaturated lactone moiety were identified.

SwissADME analysis predicted high gastrointestinal absorption and moderate lipophilicity for andrographolide. BOILED-Egg analysis suggested limited blood–brain barrier permeability, although the compound remained within the physicochemical space associated with orally bioavailable compounds (Figure 3).

The estimated oral LD_50_ was approximately 1890 mg/kg, corresponding to a prediction of low acute oral toxicity. In addition, in silico organ toxicity analysis predicted a low probability of hepatotoxicity, nephrotoxicity, and neurotoxicity.

## 3. Discussion

The present study demonstrates, for the first time, the anthelmintic activity of andrographolide against *A. cantonensis*. The compound exhibited low-micromolar activity across first-stage (L1) larvae, infective third-stage (L3) larvae, and adult worms. This multi-stage design provides complementary pharmacological information: L1 larvae represent the stage released by the definitive host and provide a convenient platform for phenotypic screening; L3 larvae constitute the infective stage responsible for host invasion and tissue migration; and adult worms, which develop in the definitive rodent host rather than ordinarily in humans, enable evaluation against a morphologically and physiologically distinct developmental stage. Activity against adults therefore demonstrates a broader stage-specific profile but should not be interpreted as direct evidence of therapeutic relevance to human neuroangiostrongyliasis. Collectively, these findings expand the known antiparasitic spectrum of andrographolide and reinforce the relevance of natural products as sources of chemically diverse scaffolds for anthelmintic drug discovery.

The potency observed for andrographolide against the larval stages of *A. cantonensis* was comparable to that previously reported for other bioactive natural products and natural product-derived compounds evaluated using the same experimental platform. For example, piplartine exhibited EC_50_ values of 8.3 and 10.4 µM against L1 and L3 larvae, respectively [26], whereas the sesquiterpene spathulenol displayed an EC_50_ of 7.6 µM against L1 larvae [27]. Likewise, the lignan cubebin showed EC_50_ values of 4.7 and 15.3 µM against L1 and L3 larvae, respectively [28], while 1,10-phenanthroline-5,6-dione derivatives exhibited EC_50_ values ranging from 6.4 to 25.2 µM against L1 larvae [29]. Taken together, these comparisons indicate that andrographolide exhibits anthelmintic activity comparable to that of other bioactive natural products evaluated against *A. cantonensis* larvae. Importantly, the present study extends this pharmacological characterization by demonstrating that andrographolide also retains potent activity against adult worms, a developmental stage not evaluated in these previous studies.

The phenotypic alterations observed following drug exposure provide a descriptive basis for future mechanistic investigation. Pyrantel, included as a phenotypic comparator, induced the characteristic coiled phenotype typically associated with cholinergic neuromuscular hypercontraction in nematodes [30]. In contrast, andrographolide-treated larvae exhibited predominantly elongated phenotypes, closely resembling those induced by albendazole. These distinct phenotypic profiles are consistent with a response different from the characteristic pyrantel-associated phenotype; however, phenotypic observations alone cannot establish or exclude a specific molecular mechanism.

Previous studies have demonstrated that andrographolide modulates multiple cellular pathways associated with oxidative stress, mitochondrial homeostasis, inflammation, and programmed cell death [16]. In other biological systems, the compound has been reported to regulate ROS production, mitochondrial function, ferroptosis-associated pathways, and PI3K/AKT/mTOR signaling [24]. Disruption of redox homeostasis and mitochondrial metabolism has emerged as an important mechanism underlying the activity of several anthelmintic compounds, including natural products active against *A. cantonensis* [13,31]. These observations provide hypotheses for future investigation in this nematode; however, none of these pathways were examined in the present study, and no mechanistic conclusion can be drawn from the current data.

Another important finding of the present study was the preliminary experimental selectivity of andrographolide. Likewise, no toxicity was observed in *C. elegans*, which was used as a complementary whole-organism invertebrate toxicity model rather than as a surrogate for parasite-specific selectivity. The difference in susceptibility between *C. elegans* and *A. cantonensis* may reflect species-specific differences in compound uptake, metabolism, or target biology, which were not investigated in the present study. Although these findings were obtained exclusively in vitro, they indicate that the antiparasitic activity of andrographolide is unlikely to result from nonspecific toxicity. This observation is particularly relevant considering the need for new anthelmintic compounds with improved safety profiles for the treatment of tissue-migrating helminth infections [32,33,34].

The favorable experimental selectivity observed in the present study was complemented by the in silico ADMET analysis. Andrographolide exhibited physicochemical characteristics compatible with drug-likeness, including compliance with the Lipinski, Ghose, Veber, Egan, and Muegge rules, together with the absence of PAINS alerts. Two Brenk alerts associated with the α,β-unsaturated lactone moiety, an electrophilic Michael acceptor, were identified. This structural feature may contribute to biological activity through covalent interactions but may also raise potential off-target reactivity concerns, which require experimental evaluation [35,36]. In addition, computational analysis predicted low probabilities of hepatotoxicity, nephrotoxicity, and neurotoxicity, together with a relatively high predicted oral LD_50_. These computational predictions require experimental validation and should be interpreted as preliminary pharmacological indicators.

An important translational consideration is the predicted blood–brain barrier permeability of andrographolide. The BOILED-Egg model predicted limited passive BBB penetration. Previous experimental studies have reported neurovascular and neuroprotective effects of andrographolide, including modulation of BBB integrity, reduction in oxidative stress, and protection of cerebral endothelial cells through PI3K/AKT/mTOR and NRF2/HO-1 signaling pathways [24]. However, effects on BBB-associated pathways do not demonstrate that pharmacologically relevant concentrations of andrographolide cross the BBB or reach the central nervous system. Future studies could explore strategies to improve CNS delivery, including structural optimization toward more lipophilic analogs and the development of nanoformulations designed to enhance brain exposure. Experimental pharmacokinetic and tissue-distribution studies will be required to determine whether such approaches improve CNS exposure, particularly considering the neurotropic nature of *A. cantonensis* infection.

Some limitations of the present study should be acknowledged. The anthelmintic activity of andrographolide was evaluated exclusively in vitro, and no mechanistic, pharmacokinetic, tissue-distribution, or in vivo efficacy studies were performed. Consequently, it remains unknown whether concentrations sufficient to reproduce the observed in vitro activity can be achieved at the sites of infection, particularly in the central nervous system. Although the compound exhibited activity against multiple developmental stages of *A. cantonensis*, including adult worms, further studies are required to determine whether this in vitro profile translates into therapeutic efficacy in experimental models of angiostrongyliasis. In addition, future investigations should explore structural optimization strategies for andrographolide. Semi-synthetic analogues have demonstrated enhanced biological activity in other experimental systems [37]; however, their activity against *A. cantonensis* has not been determined, precluding a meaningful structure–activity relationship analysis in this model. Nano- and liposomal formulations also represent promising approaches to improve the bioavailability and antiparasitic efficacy of andrographolide, as previously demonstrated in experimental models [17,18]. Finally, andrographolide was isolated from a single batch of *C. schoenanthus* leaves. Future studies should assess the reproducibility of its content and isolation yield across different batches and geographical origins of the plant material [38].

## 4. Materials and Methods

### 4.1. General Experimental Procedures

NMR spectra were recorded on a Bruker Ascend Evo 600 spectrometer (Billerica, MA, USA) operating at 600 MHz for ^1^H and 150 MHz for ^13^C, using CD_3_OD as solvent. The HR-ESI-MS spectrum was acquired in positive-ion mode on a Bruker Daltonics MicroTOF QII spectrometer (Billerica, MA, USA). Optical rotation values were measured in a JASCO P-2000 polarimeter (Tokyo, Japan), with Na filter (λ = 589 nm). Column chromatography was carried out using silica gel 60 (Merck, Darmstadt, Germany), while silica gel 60 PF_254_ (Merck, Darmstadt, Germany) was employed for thin-layer chromatography (TLC). HPLC analyses were performed on a Thermo Scientific Ultimate 3000 system (Waltham, MA, USA) equipped with a DAD detector (UVD-170 model, Thermo Scientific, Waltham, MA, USA) and a Kinetex^®^ C18 analytical column (Phenomenex, Torrance, CA, USA) (5 μm, 250 × 4.6 mm), using MeOH:H_2_O (1% formic acid) (65:35) as the mobile phase at a flow rate of 1.0 mL/min.

### 4.2. Plant Material

Leaves of *Cymbopogon schoenanthus* were collected in Campinas, São Paulo State, Brazil, in February 2025, from the same specimen previously studied [19]. The botanical material was identified using voucher specimen SP042740, deposited at the Herbarium of the Institute of Biosciences, University of São Paulo (IB-USP, Brazil).

### 4.3. Extraction and Isolation of Andrographolide

Dried and powdered leaves of *C. schoenanthus* (100 g) were sequentially extracted with hexanes (6 × 200 mL). Removal of the solvent under reduced pressure afforded the hexane extract (1.4 g), which was subjected to silica gel column chromatography and eluted with hexane:EtOAc (6:4, 3:7 and 1:1), followed by 100% EtOAc, yielding seven groups (A–G). Part of group G (256 mg) was further purified by silica gel column chromatography using hexane:EtOAc (1:1) as eluent to afford andrographolide (58.5 mg).

*Andrographolide*. White amorphous solid. (99% purity by HPLC). [α]_D_^20^—95 (*c* 0.60 MeOH). ^1^H NMR (600 MHz, CD_3_OD): δ/ppm 6.85 (t, *J* = 6.8 Hz, H-12), 5.01 (d, *J* = 6.0 Hz, H-14), 4.88 (s, H-17a), 4.66 (s, H-17b), 4.46 (dd, *J* = 10.2 and 6.1 Hz, H-15a), 4.14 (m, H-15b and H-19a), 3.41–3.36 (m, H-3), 3.31 (s, H-19), 2.63–2.52 (m, H-11), 2.42 (m, H-7a), 2.03 (m, H-7b), 1.92 (m, H-9), 1.89–1.75 (m, H-1a, H-2, and H-6a), 1.43–1.26 (m, H-1, H-5, and H-6b), 1.22 (s, H-18), 0.75 (s, H-20). ^13^C NMR (150 MHz, CD_3_OD): δ/ppm 171.2 (C-16), 147.9 (C-12), 147.4 (C-8), 128.4 (C-13), 107.8 (C-17), 79.5 (C-3), 74.7 (C-15), 65.2 (C-14), 63.6 (C-19), 56.0 (C-9), 54.9 (C-5), 42.3 (C-4), 38.6 (C-10), 37.6 (C-7), 36.7 (C-1), 27.6 (C-2), 24.3 (C-11), 23.8 (C-6), 22.0 (C-18), 14.1 (C-20). HR-ESI-MS *m*/*z* 373.1988 [M + Na]+ (calcd. for C_20_H_30_O_5_Na 373.1991).

### 4.4. Parasites and Cell Lines

The life cycle of *Angiostrongylus cantonensis* (NPDN-AC strain) was maintained at the Research Center for Neglected Diseases (NPDN), Guarulhos University, Brazil, using *Biomphalaria glabrata* as the intermediate host and *Rattus norvegicus* as the definitive host, as previously described [5]. First-stage (L1) larvae, infective third-stage (L3) larvae, and adult worms obtained from this life cycle were used in the in vitro anthelmintic assays.

African green monkey kidney epithelial (Vero; ATCC CCL-81) and human neuroblastoma (SH-SY5Y; BCRJ Code 0223) cell lines were obtained from the American Type Culture Collection (ATCC, Manassas, VA, USA) and the Rio de Janeiro Cell Bank (BCRJ, Rio de Janeiro, Brazil), respectively. Cells were cultured in Dulbecco’s Modified Eagle Medium (DMEM) supplemented with 10% heat-inactivated fetal bovine serum, 100 U/mL penicillin, and 100 μg/mL streptomycin at 37 °C in a humidified atmosphere containing 5% CO_2_ [39].

### 4.5. Anthelmintic Activity Against L1 and L3 Larvae

First-stage (L1) larvae were recovered from the feces of infected *R. norvegicus*, whereas infective third-stage (L3) larvae were isolated from the tissues of infected *B. glabrata* by artificial digestion, as previously described [5]. The in vitro anthelmintic activity of andrographolide against first-stage (L1) and infective third-stage (L3) larvae of *A. cantonensis* was evaluated using a previously established motility-based phenotypic assay [5,6,7]. Approximately 50 larvae were transferred to each well of 96-well culture plates containing 200 µL of RPMI 1640 medium containing 100 U/mL penicillin and 100 µg/mL streptomycin.

Albendazole and pyrantel pamoate were obtained from *Ecovet Indústria Veterinária Ltd.* (São Paulo, SP, Brazil), both with 99% purity. Andrographolide, albendazole (reference drug), and pyrantel pamoate (phenotypic comparator) were dissolved in DMSO and initially screened at 100 μM. Concentration–response assays were subsequently performed for all three compounds using 3-fold serial dilutions starting from 100 μM for EC_50_ determination. Parasites incubated in culture medium containing 0.5% DMSO served as the vehicle control, corresponding to the maximum solvent concentration present in any treatment well. After 24 h of incubation, larval motility was evaluated under an inverted microscope using a four-point scoring system: 1, immotile; 2, intermittent movement; 3, slow movement; and 4, highly active [26,28].

Each larva was classified individually. Viability was calculated directly from the proportion of immotile larvae using the formula: viability (%) = [1 − (number of score 1 larvae/total number of larvae evaluated)] × 100. A predominant well-level motility score was assigned when at least 60% of the larvae were classified within the same category. Assessments were performed without blinding by two experienced observers who simultaneously examined real-time images projected on a monitor from a camera-equipped microscope, and classifications were assigned by consensus. Each concentration was tested in three wells, corresponding to approximately 150 larvae per experiment, and the assay was independently repeated three times.

### 4.6. Anthelmintic Activity Against Adult Worms

Adult *A. cantonensis* worms were recovered from the lungs of infected *R. norvegicus* and immediately transferred to 12-well culture plates containing one male and one female worm per well in 2 mL of RPMI 1640 medium containing 100 U/mL penicillin, 100 µg/mL streptomycin, and 5% heat-inactivated fetal bovine serum. Andrographolide, albendazole, and pyrantel pamoate were initially screened at 100 μM, followed by 3-fold serial dilutions for EC_50_ determination. The vehicle control consisted of worms incubated in the same culture medium with 0.5% DMSO, corresponding to the maximum solvent concentration used in treated wells. Plates were incubated at 37 °C in a humidified atmosphere containing 5% CO_2_ for 24 h. Parasite viability was evaluated under a stereomicroscope according to the same motility scoring system and assessment procedure described for the larval assays, and worms showing no spontaneous movement for at least 2 min were classified as nonviable for the purposes of the assay. Three distinct worm pairs were evaluated per concentration in each experiment, and the assay was independently repeated three times using different worm pairs.

### 4.7. Cytotoxicity Assay

The cytotoxicity of andrographolide was evaluated in Vero and SH-SY5Y cell lines using the MTT colorimetric assay [40], as previously described [41,42]. Cells were seeded in 96-well culture plates and allowed to adhere for 24 h before treatment. Andrographolide was tested using 3-fold serial dilutions starting from 500 μM. Following 48 h of incubation at 37 °C in a humidified atmosphere containing 5% CO2, MTT solution was added to each well and incubated for an additional 4 h. The resulting formazan crystals were dissolved in DMSO, and absorbance was measured at 570 nm using a microplate reader, Epoch Microplate Spectrophotometer (BioTek Instruments, Winooski, VT, USA). Cell viability was expressed relative to untreated controls [43].

### 4.8. Toxicity Assessment in C. elegans

The toxicity of andrographolide was evaluated in the free-living nematode *C. elegans* (N2 strain) as a complementary whole-organism invertebrate toxicity model, as previously described [44]. Synchronized L4 larvae were transferred to 96-well culture plates containing M9 buffer supplemented with *Escherichia coli* OP50 as a food source. Andrographolide was tested using 3-fold serial dilutions starting from 1000 μM, and nematodes were incubated for 24 h at 20 °C. Nematode viability was assessed under an inverted microscope based on spontaneous movement and responsiveness to gentle mechanical stimulation [42]. Each concentration was tested in triplicate, and all experiments were independently repeated three times.

### 4.9. In Silico ADMET and Drug-Likeness Analysis

The pharmacokinetic properties and drug-likeness of andrographolide were predicted using the SwissADME web server (https://www.swissadme.ch/, accessed on 17 February 2026) [45]. Physicochemical descriptors, gastrointestinal absorption, blood–brain barrier permeability, and compliance with the Lipinski, Ghose, Veber, Egan, and Muegge drug-likeness rules were evaluated. Structural alerts, including PAINS and Brenk filters, were also analyzed. The BOILED-Egg model was used to predict passive gastrointestinal absorption and blood–brain barrier permeability [46].

Acute oral toxicity (LD_50_), Globally Harmonized System (GHS) toxicity classification, and organ-specific toxicity endpoints, including hepatotoxicity, nephrotoxicity, and neurotoxicity, were predicted using the ProTox-3.0 web server (https://tox.charite.de/, accessed on 18 February 2026) [47].

### 4.10. Statistical Analysis

All assays were conducted as three independent experiments (*n* = 3), each comprising three technical replicates. For the anthelmintic assays, concentration–response curves were fitted separately for each independent experiment by nonlinear regression using the built-in EC_50_ shift model, with X entered as log_10_(concentration), in GraphPad Prism version 10.0 (San Diego, CA, USA) [48]. Top, bottom, and Hill slope parameters were estimated freely, without fixed constraints at 0% or 100%. Prism generated asymptotic 95% confidence intervals for each individual curve fit. EC_50_ values were calculated separately for each independent experiment and subsequently expressed as mean ± standard deviation (SD) of the three independently determined estimates. EC_50_ values were compared among compounds separately for each developmental stage using one-way ANOVA followed by Tukey’s multiple-comparisons test. Differences were considered statistically significant at *p* < 0.05 [49].

## 5. Conclusions

Andrographolide demonstrated consistent in vitro anthelmintic activity across multiple developmental stages of *A. cantonensis*, together with preliminary selectivity in mammalian cells and *C. elegans* and predicted drug-like properties. These findings support the prioritization of andrographolide as a promising in vitro anthelmintic hit for further preclinical investigation and reinforce the value of natural products as sources of chemically diverse anthelmintic scaffolds. Further studies are required to establish its mechanism of action, pharmacokinetic properties, CNS exposure, and therapeutic efficacy in vivo before its potential for the treatment of angiostrongyliasis can be determined.

## Figures and Tables

**Table 1:** In vitro anthelmintic activity of andrographolide against first-stage (L1) larvae, infective third-stage (L3) larvae, and adult worms of *Angiostrongylus cantonensis*.

Compound	*A. cantonensis* EC_50_ (µM)		
	L1	L3	Adult
Andrographolide	12.5 ± 1.6	11.2 ± 1.5	7.3 ± 2.1
Albendazole	15.1 ± 2.2	15.6 ± 1.9	6.8 ± 1.8
Pyrantel pamoate	11.3 ± 1.4	16.6 ± 2.7	19.4 ± 2.3 **

EC_50_ values were determined for L1 larvae, L3 larvae, and adult worms after 24 h of incubation. Data are presented as mean ± standard deviation (SD) from three independent experiments. ** *p* < 0.01 versus andrographolide and albendazole for adult worms, as determined by one-way ANOVA followed by Tukey’s multiple-comparisons test.

**Table 2:** Toxicity in mammalian cells and *Caenorhabditis elegans* and selectivity index of andrographolide and reference drugs.

Compound	Mammalian Cells CC_50_ (µM)		Selectivity Index			*C. elegans* LD_50_ (µM)
	Vero	SH-SY5Y	L1	L3	Adults	
Andrographolide	>500	>500	>40	>44	>68	>1000
Albendazole	>500	>500	>33	>32	>73	14.6 ± 1.8
Pyrantel pamoate	>500	>500	>44	>30	>25	26.2 ± 2.7

Toxicity in mammalian cells is expressed as CC_50_ values, whereas toxicity in *C. elegans* is expressed as LD_50_ values. The selectivity index (SI) was calculated as the ratio between CC_50_ and EC_50_ values. Because CC_50_ values were not reached at the highest concentration tested, the corresponding SI values represent minimum (lower-bound) estimates.

## Data Availability

The data supporting the findings of this study are available within the article and its Supplementary Materials. Further information is available from the corresponding author upon request.

## Supplementary Materials

The following supporting information can be downloaded at https://www.mdpi.com/article/10.3390/ph19091435/s1, Figure S1: HPLC analysis for andrographolide; Figure S2: UV spectrum for andrographolide; Figure S3: ESI-HRMS (positive mode) spectrum for andrographolide; Figure S4: ^1^H NMR spectrum of andrographolide (δ, CD_3_OD, 600 MHz); Figure S5: ^13^C NMR spectrum of andrographolide (δ, CD_3_OD, 150 MHz).

## Abbreviations

The following abbreviations are used in this manuscript:

ADME	Absorption, Distribution, Metabolism, and Excretion
BBB	Blood–Brain Barrier
CC_50_	Cytotoxic Concentration at 50%
CD_3_OD	Deuterated Methanol
d	Doublet
dd	Doublet-Doublet
DMEM	Dulbecco’s Modified Eagle Medium
DMSO	Dimethyl sulfoxide
EC_50_	Effective Concentration at 50%
EtOAc	Ethyl Acetate
HIA	Passive Gastrointestinal Absorption
HPLC	High-Performance Liquid Chromatography
L1	First-Stage Larvae
L3	Infective Third-Stage Larvae
LD_50_	Lethal Dose 50%
MeOH	Methanol
MHz	Megahertz
NMR	Nuclear Magnetic Resonance
PAINS	Pan-Assay Interference
RPMI	Roswell Park Memorial Institute
TLC	Thin Layer Chromatography
