# Supplementary material for: Andrographolide as a Promising Diterpenoid Scaffold Against the Neurotropic Nematode Angiostrongylus cantonensis
Source: Pharmaceuticals (Basel). 2026 Sep 10;19(9):1435. doi: 10.3390/ph19091435 (PMC13610970; doi:10.3390/ph19091435)
Supplement: Supplementary file 1 [file pharmaceuticals-19-01435-s001.zip › pharmaceuticals-4476067-supplementary.pdf]

Andrographolide as a Promising Diterpenoid Scaffold Against the Neurotropic Nematode *Angiostrongylus cantonensis*

Fabiana R. S. Tominaga [1], Davi Luna-Tavares [1], Lucas Fukui-Silva [2], Thainá R. Teixeira [2], Henrique Barbosa [3], João Henrique G. Lago [3,*] and Josué de Moraes [1,2,*]

[1] Research Center on Neglected Diseases, Brazil University, São Paulo 08230-030, SP, Brazil; fabitominaga@gmail.com (F.R.S.T.); davilunatav@gmail.com (D.L.-T.)
[2] Research Center on Neglected Diseases, Guarulhos University, Guarulhos 07023-070, SP, Brazil; fukui.npdn@gmail.com (L.F.-S.); thainanpdn@gmail.com (T.R.T.)
[3] Center for Natural and Human Sciences, Federal University of ABC, São Paulo 09210-180, SP, Brazil; heinrichbarbosa@gmail.com
* Correspondence: joao.lago@ufabc.edu.br (J.H.G.L.); josue.moraes@prof.ung.br or moraesnpdn@gmail.com (J.d.M.)

SUPPLEMENTARY MATERIAL

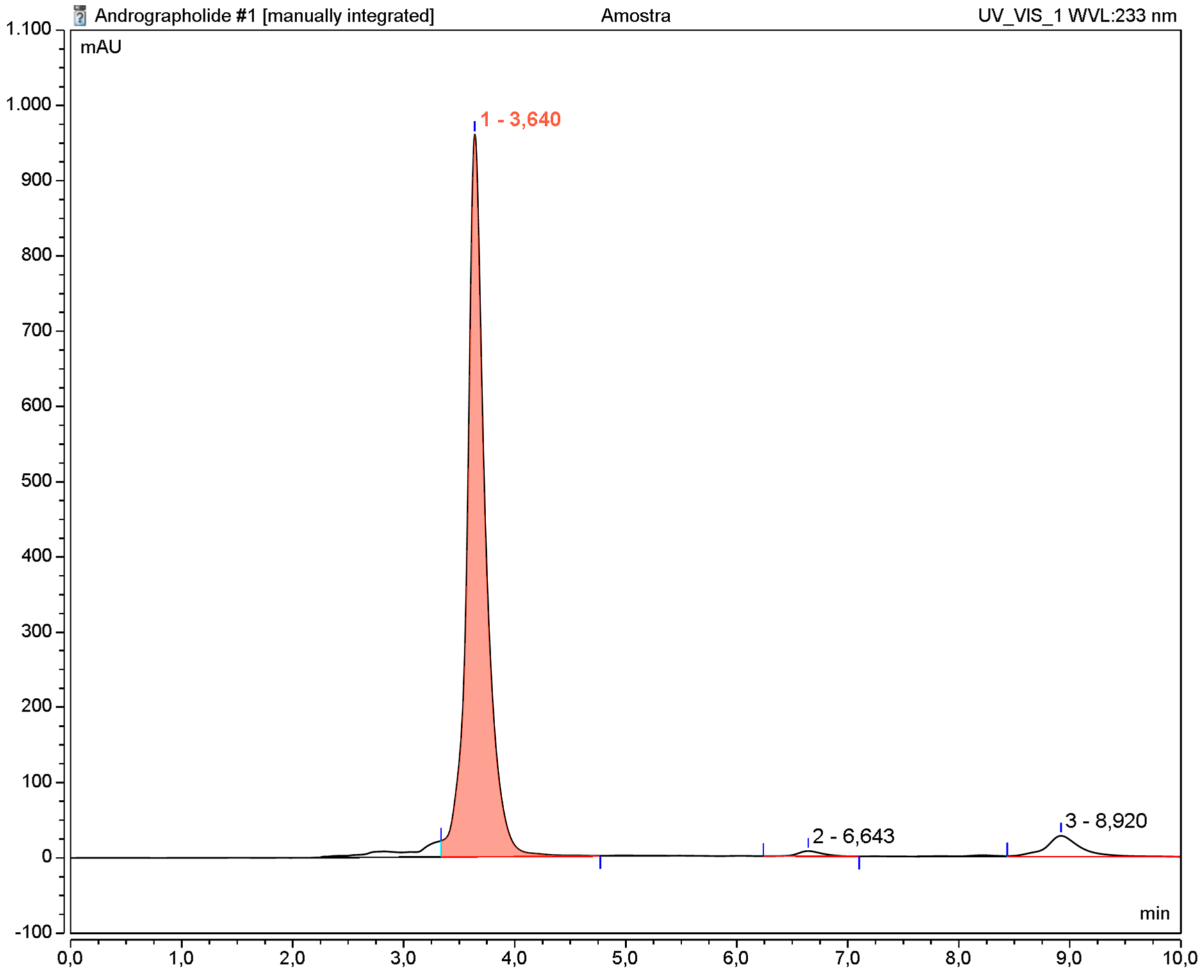

Figure S1. HPLC analysis for andrographolide, performed using a Thermo Scientific Ultimate 3000 system equipped with a DAD detector (UVD-170 model) and a Kinetex® C18 analytical column (5 µm, 250 × 4.6 mm), using MeOH:H_2O (1% formic acid) (65:35) as the mobile phase at a flow rate of 1.0 mL/min.

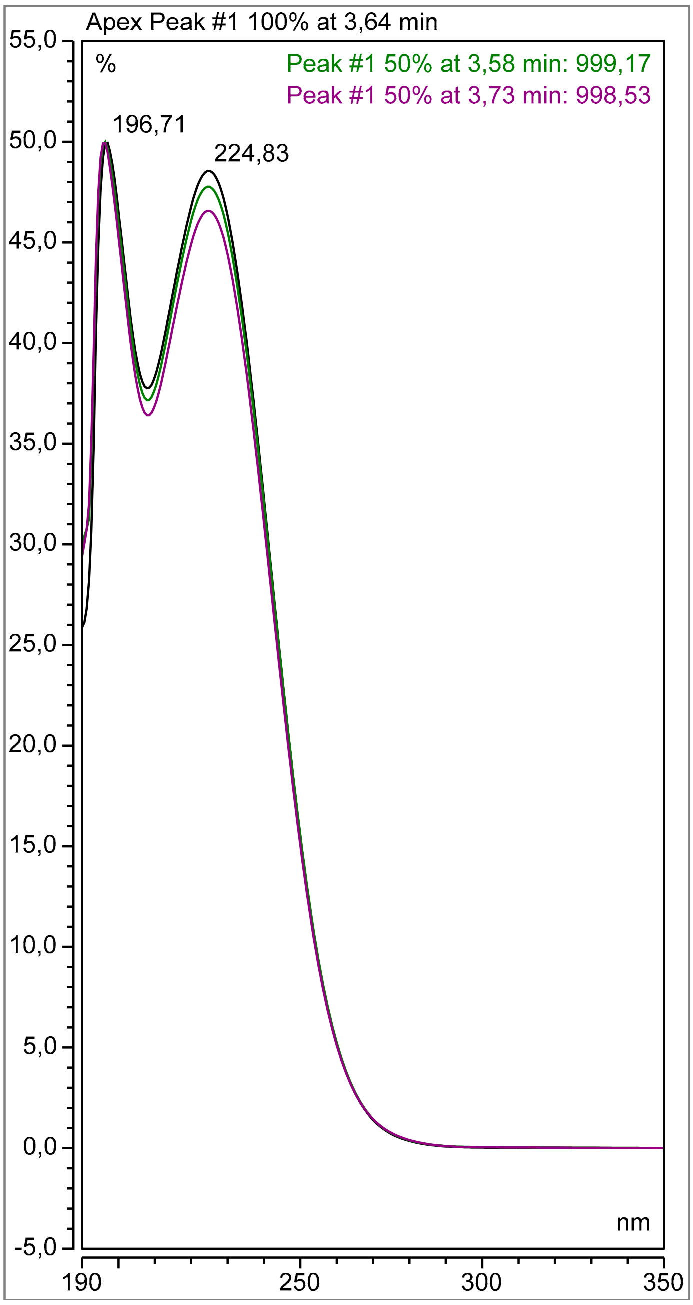

Figure S2. UV spectrum for andrographolide.

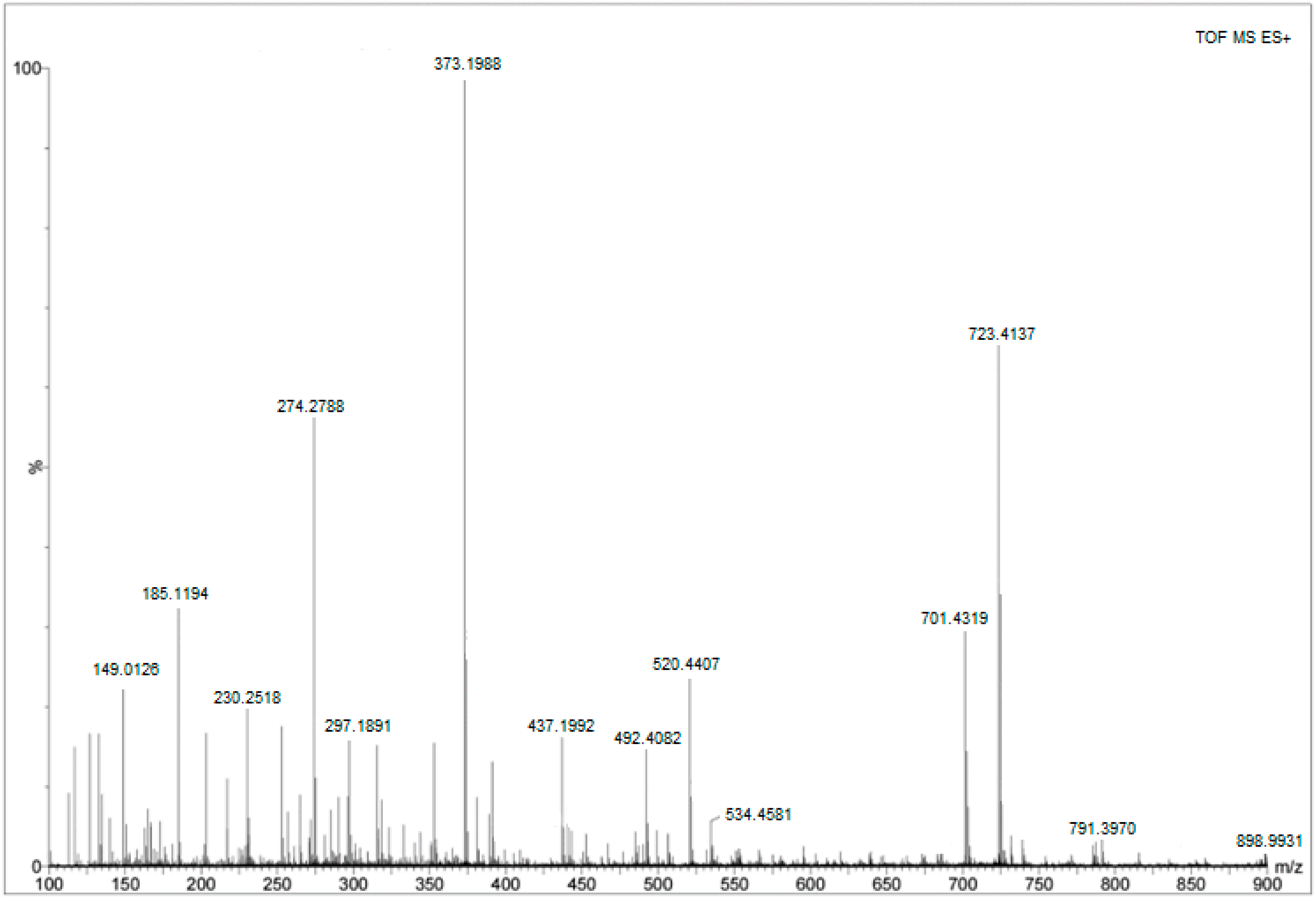

Figure S3. ESI-HRMS (positive mode) spectrum for andrographolide.

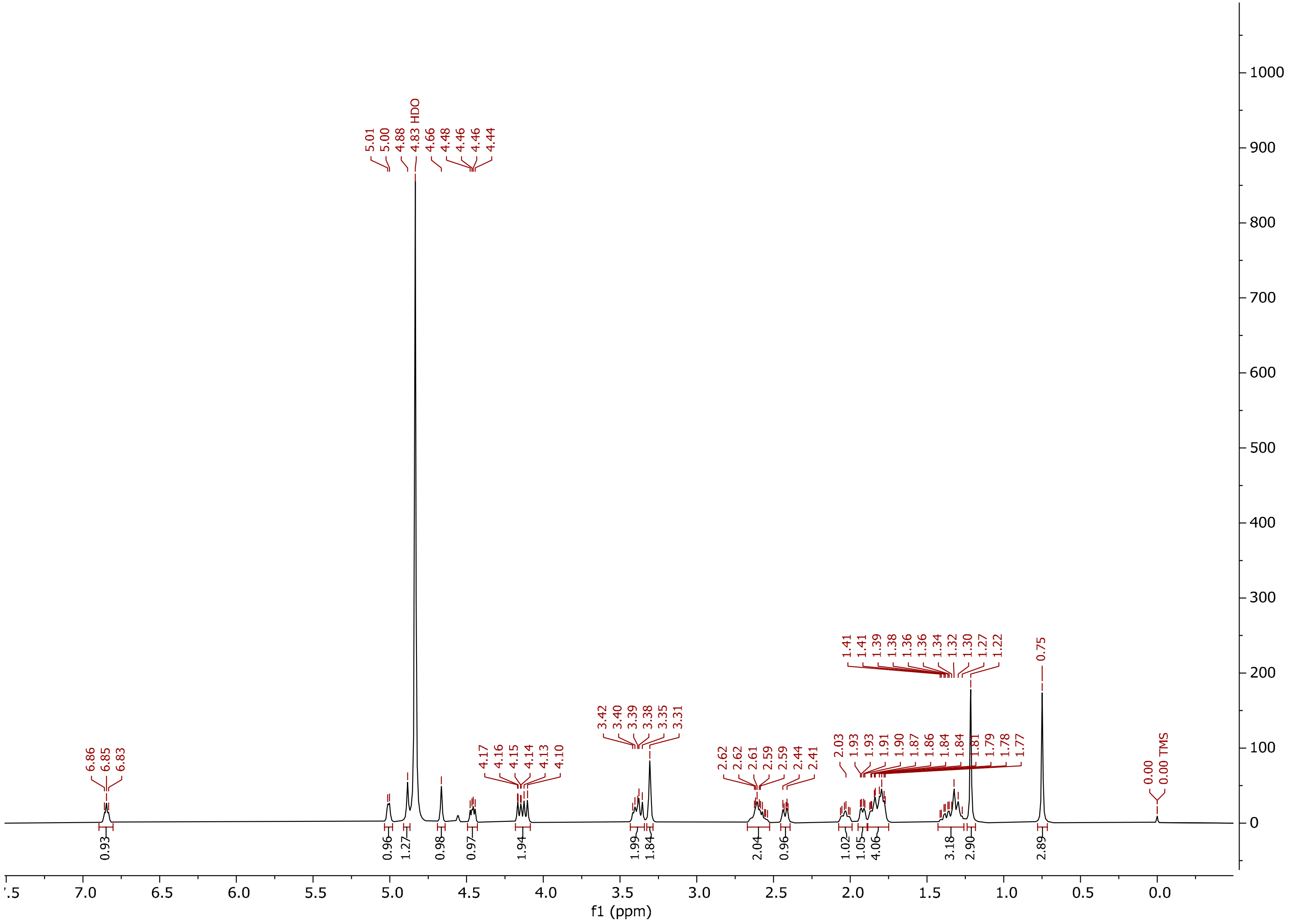

Figure S4. ^{1}H NMR spectrum of andrographolide (δ, CD_3OD, 600 MHz)

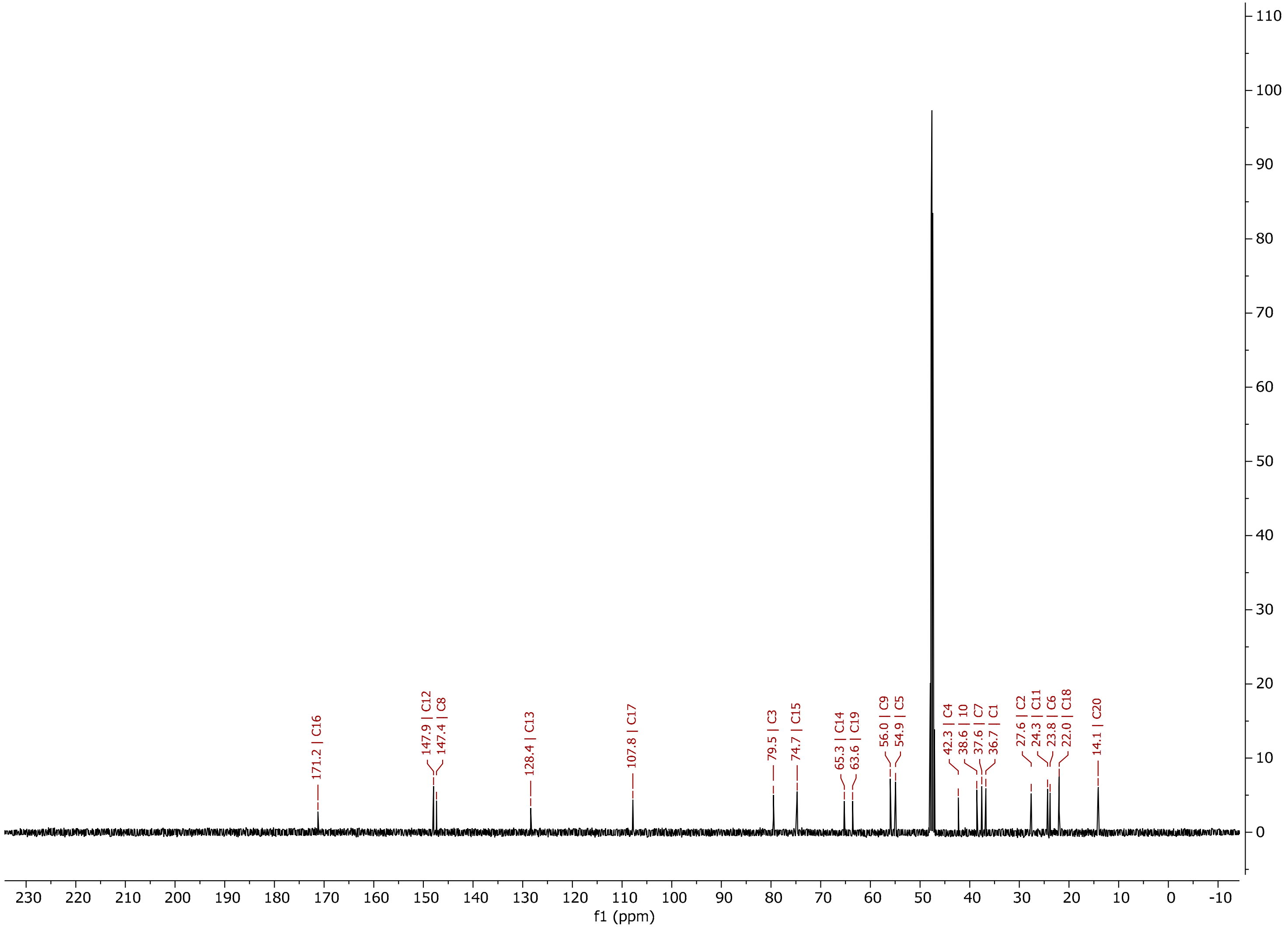

Figure S5. ^{13}C NMR spectrum of andrographolide (δ, CD_3OD, 150 MHz)
